# Synthesis and Characterization of Graphene/ITO Nanoparticle Hybrid Transparent Conducting Electrode

**DOI:** 10.1007/s40820-017-0174-0

**Published:** 2017-11-28

**Authors:** Bastian Waduge Naveen Harindu Hemasiri, Jae-Kwan Kim, Ji-Myon Lee

**Affiliations:** Department of Printed Electronics Engineering, Sunchon National University, Suncheon, Jeonnam 57922 South Korea

**Keywords:** Graphene, ITO nanoparticles, Electroless deposition, Crystallization, Raman

## Abstract

The combination of graphene with conductive nanoparticles, forming graphene–nanoparticle hybrid materials, offers a number of excellent properties for advanced engineering applications. A novel and simple method was developed to deposit 10 wt% tin-doped indium tin oxide (ITO) nanoparticles on graphene. The method involved a combination of a solution-based environmentally friendly electroless deposition approach and subsequent vacuum annealing. A stable organic-free solution of ITO was prepared from economical salts of In(NO_3_)_3_^·^H_2_O and SnCl_4_. The obtained ITO nanostructure exhibited a unique architecture, with uniformly dispersed 25–35 nm size ITO nanoparticles, containing only the crystallized In_2_O_3_ phase. The synthesized ITO nanoparticles–graphene hybrid exhibited very good and reproducible optical transparency in the visible range (more than 85%) and a 28.2% improvement in electrical conductivity relative to graphene synthesized by chemical vapor deposition. It was observed that the ITO nanoparticles affect the position of the Raman signal of graphene, in which the D, G, and 2D peaks were redshifted by 5.65, 5.69, and 9.74 cm^−1^, respectively, and the annealing conditions had no significant effect on the Raman signatures of graphene.

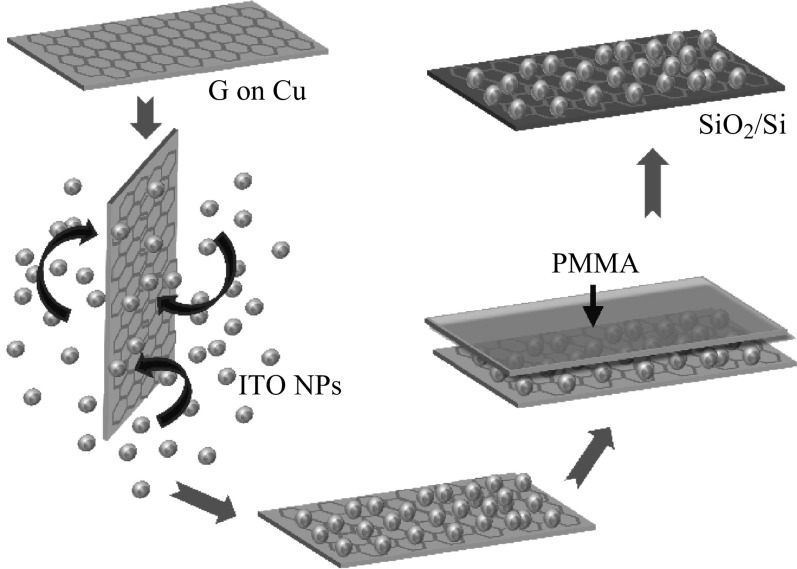

## Highlights

- A green, simple, and novel approach was used for hybridization of chemical vapor deposition (CVD) graphene with 10 wt% tin-doped ITO nanoparticles.
- A unique architecture with monodisperse 23–35 nm ITO nanoparticles over graphene was constructed to form a stable organic-free solution of ITO.
- High improvements on optoelectrical properties with 28.2% in electrical conductivity relative to individual CVD graphene and more than 85% optical transmittance in the visible range were achieved.

## Introduction

Graphene is an ideal two-dimensional (2D) carbon allotrope with an *sp* ^2^ honeycomb structure and has attracted attention since it was conclusively isolated from graphite in 2004 via an exfoliation method [1–7]. Owing to its unique properties such as having charge carriers that mimic massless relativistic Dirac fermions, a weak optical absorptivity (2.3%), exhibiting the anomalous quantum Hall effect, superior thermal conductivity, vanishing hyperfine interaction features, and unusually high mechanical strength, graphene is a promising material for a variety of applications, such as nanoelectronic devices, transparent conductive films, transistors, chemical and biochemical sensors, actuators, clean energy devices, flexible optoelectronic devices, and energy and data storage technologies [2, 4, 8–13].

A range of approaches have been attempted, and various theoretical studies have been performed recently to ensure the real-world application of graphene with a variety of advantages, rather than using graphene alone, such as multilayer graphene sheets that can form hybrid composites with other conductive materials and decoration of graphene with metal and metal oxide nanoparticles [14–20]. The combination of graphene with conductive nanomaterials such as metal nanoparticles has led to the recent development of graphene–metal–nanoparticle hybrid structures aimed at additional improvement and manipulation of both the electronic and magnetic properties of graphene [21–29]. However, metal nanoparticles show weak interaction with carbon, causing high agglomeration over carbon materials with a low active surface area, resulting in performance degradation during continuous long-term operation. Simultaneously, metal oxides have recently been used as a supporting material for metal particles, because several conductive metal oxides are catalyst supports in electrocatalysis, which show excellent mechanical strength and high thermal stability in an oxidizing environment, in which graphene cannot survive [30, 31].

Indium tin oxide or tin-doped indium oxide (ITO) is recognized as a viable semiconducting metal oxide with high conductivity, chemical stability, good optical transparency, and excellent substrate adherence, making it a prime candidate in most advanced electronic applications [32, 33]. The incorporation of ITO with graphene has attracted considerable attention for the development of hybrid materials. This hybrid material shows superior electrical conductivity over those of the intrinsic materials, owing to the enhancement of both the surface carrier charge mobility and carrier density of the hybrid material when graphene was incorporated with ITO [34]. ITO acts as an electron dopant, which enhances the surface carrier density of graphene. Furthermore, graphene–ITO nanoparticle structures show superior electrocatalytic activity, long-term stability, and potentially allow the optimization of dispersion as a metal electrocatalyst support. The interaction between metal particles and metal oxides is better than that between metal particles and graphene, such that ITO acts as a stabilizer that improves the existence and durability of the metal catalysts in graphene [30]. Several new processes were proposed and developed recently to synthesize graphene-based metal oxide nanocomposite and hybrid materials, such as chemical reduction, electrochemical synthesis, a microwave-assisted method, solvothermal and hydrothermal methods, electroless deposition, and a solution-based dipping method. It is important to develop a method to deposit ITO nanoparticles over graphene with no or low defect density between ITO nanoparticles and graphene and without damaging the underlying graphene, because the existence of graphene with a lower limit of defects is essential for graphene-based applications [35]. Although a solution-based electroless deposition has been used to decorate graphene with metal nanoparticles, to the best of the authors’ knowledge, the deposition of ITO nanoparticles on graphene through a solution-based method has not been reported.

In this study, we present a novel, simple, and versatile method for the deposition of ITO nanoparticles on graphene grown by chemical vapor deposition (CVD) method through a combination of a solution-based electroless deposition method and subsequent annealing. The solution used as the ITO source was prepared using an environmentally friendly aqueous sol–gel and the concentration of Sn was adjusted to 10 wt% in the final ITO nanoparticles.

## Experimental Section

### Synthesis of Aqueous ITO Sol–Gel

Aqueous ITO sol–gel was synthesized using In(NO_3_·*x*H_2_O (99.99% trace metals basis, Aldrich) and SnCl_4_ (99.995% trace metals basis, Aldrich) as initial materials. The stoichiometry of In(NO)_3_·*x*H_2_O was determined by thermogravimetric analysis, prior to mixing the materials, and hydration number was obtained as one. The materials were mixed using the appropriate stoichiometry as follows to obtain Sn 10 wt% for the final ITO solution. 3.876 g of In(NO)_3_·*x*H_2_O and 0.648 g (291.1 μL) of SnCl_4_ were mixed in 15 mL of deionized (DI) water, and the resultant solution was refluxed at 40 °C for 2 h. The obtained sol was aged for 48 h to achieve gelation before application.

### Synthesis of CVD Graphene

**Fig. 1:**
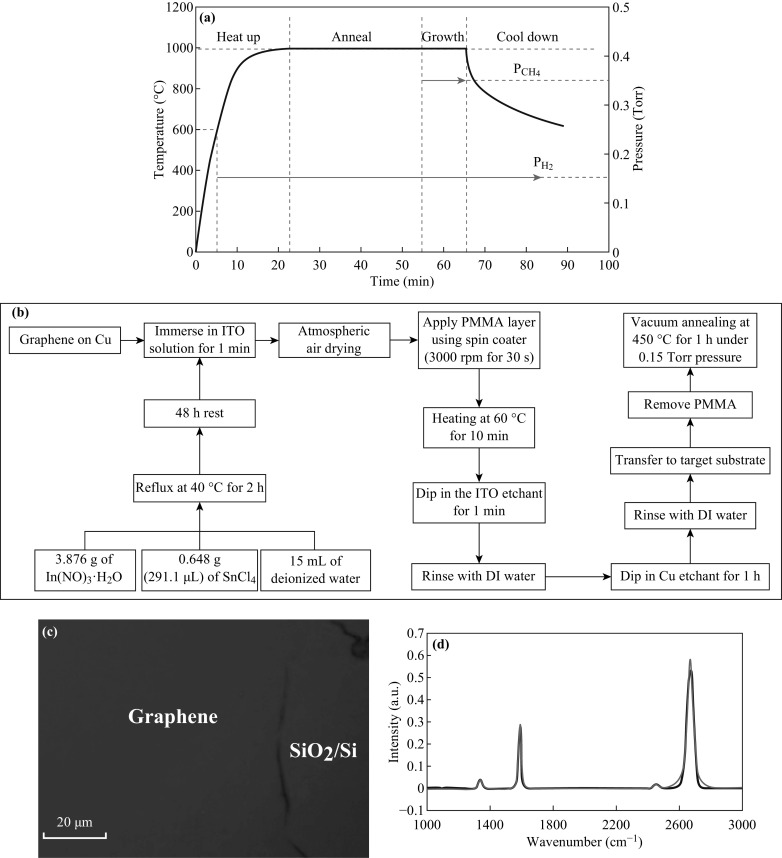
**a** Schematic of the CVD graphene growth process. **b** Process flow chart of the synthesis of ITO nanoparticles/graphene hybrids. **c** Optical microscopic image of graphene on SiO_2_ (300 ± 5 nm)/Si substrate. **d** Raman spectrum of CVD graphene (excitation wavelength of 532 nm) on SiO_2_ (300 ± 5 nm)/Si (black line) and Lorentzian fitted curve (red line). (Color figure online)

Over 99.99% pure and 25-µm-thick Cu foil was used as a catalytic substrate to synthesize graphene through the CVD method. The foil was cut into desired sizes (4 × 5 cm^2^) and washed ultrasonically with acetone, isopropanol, and DI water, and dried using N_2_ gas flow. The cleaned Cu foil was loaded in the CVD chamber and heated to 1000 °C. 99.999% pure H_2_ gas was introduced to the chamber at 600 °C under a flow rate of 15 sccm, and the chamber pressure was maintained at 0.15 ± 0.01 Torr. Cu strips were annealed at 1000 °C for 30 min prior to introducing CH_4_. High-quality pure CH_4_ gas was supplied to the chamber with a 30 sccm flow rate along with a continuous supply of H_2_ gas, and the chamber pressure was maintained at 0.5 ± 0.1 Torr. The supply of both gases was stopped after 10 min of CH_4_ gas supply, after which the heater system was turned off, and the chamber system was cooled to room temperature (shown in Fig. 1a).

### Decoration of Graphene with ITO Nanoparticles

Graphene-grown Cu strips were immersed in ITO solution for 1 min and dried in an atmospheric condition for 30 min. The rate at which the graphene-grown Cu strips were dipped or pulled vertically through the solution was precisely controlled and kept constant at a very low value. The dried samples were transferred to SiO_2_ (300 ± 10 nm)/Si substrates using a poly(methyl methacrylate) (PMMA)-based indirect transfer method as follows: PMMA solution was prepared by mixing 4.5 g of PMMA ((C_5_O_2_H_8_)_*n*_, average *M*_w ~_ 120,000, Aldrich) with 41 mL of monochlorobenzene (C_6_H_5_Cl, 1.11 g cm^−3^, Aldrich) and stirred at 80 °C for 1 h to obtain a clear solution. PMMA was coated on the samples using a spin coater at 3000 rpm for 30 s and heated at 60 °C for 10 min. The rear sides of the PMMA-coated samples were dipped in the ITO etchant (LCE-12 K) for 1 min, followed by rinsing with DI water and in a metal etchant for 5 min, then rinsing with DI water to remove the dried ITO solution and graphene on the rear side of the Cu strips. The samples were transferred to the 50% diluted metal etchant solution (Transene CE100, DI water/etchant = 1:2) for 1 h to completely remove the Cu foil. The Cu-removed samples were transferred to HCl 10% solution, held for 5 min to remove contaminants from samples, and then transferred to DI water for 10 min to remove any residuals. The SiO_2_/Si (target substrate) was chemically and ultrasonically washed with acetone (5 min), isopropanol (5 min), and DI water (10 min), and dried using N_2_ gas flow. The well-cleaned samples were then transferred to SiO_2_/Si substrates. The PMMA layer was removed from the samples using acetone, 2-iso propanol, and DI water, which were then dried under N_2_ gas flow. Finally, the samples were annealed at 450 °C for 1 h under vacuum (0.15 Torr) to obtain a crystalline ITO nanostructure on the graphene. A SiO_2_ (300 ± 5 nm)/Si substrate was used as a standard reference substrate for the Raman characterization of graphene (shown in Fig. 1b).

### Characterization of the ITO Nanoparticle-Decorated Graphene

X-ray diffraction (XRD, X’Pert PRO MPD, PANalytical B.V.) was conducted with a monochromatic Cu anode (K_*α*_ radiation, wavelength 1.54060 Å) for crystallization analysis. Detailed information about the chemical and elemental composition of ITO nanoparticle-decorated graphene was determined by X-ray photoelectron spectroscopy (XPS, K-Alpha, Thermo Fisher Scientific Co. Ltd.) and energy-dispersive X-ray (EDX) spectroscopy coupled with field-emission scanning electron microscopy (FE-SEM, S-4800, HITACHI). Raman spectra (NRS-2100, Jasco) were recorded using a 532-nm excitation laser from 1000 to 3000 cm^−1^. The microstructure of the ITO nanoparticle-decorated graphene was observed with an optical microscope (Eclipse LV150, Nikon) and FE-SEM.

## Results and Discussion

### Raman Characteristics of Synthesized Graphene

Figure 1c shows optical microscopic imagery of the synthesized graphene after being transferred to the SiO_2_/Si substrate. The Raman spectrum of graphene on SiO_2_/Si is presented in Fig. 1d, showing the G band at 1589.21 cm^−1^ and 2D band at 2679.97 cm^−1^. The G band, related to the doubly degenerate phonon mode at the Brillouin zone (BZ) center, is the main Raman characteristic feature, whereas the 2D band is a second-order Raman characteristic feature originating from the double-resonance scattering process near the *K* or *K* ^′^ point for all *sp* ^2^ carbons [36]. The intensity ratio between the G and 2D peaks (*I*_2D_/*I*_G_) was obtained as 2.09. Furthermore, the peak position, shape of the peaks, and *I*_2D_/*I*_G_ ratio can be used to represent the number of layers in graphene. As shown by the red line in Fig. 1d, the 2D band exhibiting a symmetric nature and a single Lorentzian feature with a measured full width at half maximum (FWHM) of 26 cm^−1^ represents the existence of monolayer graphene. This nature of the single Lorentzian peak is evident in the monolayer graphene, because only one Raman scattering cycle is excited near the *K* and *K* ^′^ points in the BZ, revealing the single *π* electron valence band and *π* ^*^ conduction band structure [37]. A low-intensity D band, representing the disorder or defective nature of graphene, related to the breaking of carbon hexagons, was observed at 1347.66 cm^−1^ with a determined D-to-G intensity ratio (*I*_D_/*I*_G_) of 0.121, indicating a small number of intrinsic defects in the CVD-synthesized graphene.

The high intensity of the 2D band with respect to the G band, the FWHM of the 2D band, and the low intensity of the D band with very low *I*_D_/*I*_G_ demonstrate that CVD-synthesized graphene has a high-quality monolayer nature.

### ITO Nanoparticle Introduction on Graphene

**Fig. 2:**
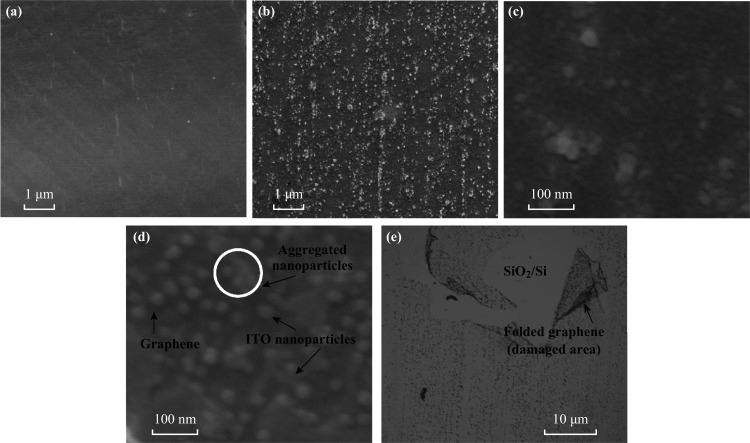
FE-SEM images of **a** CVD graphene on copper. **b** ITO nanoparticle-deposited (1-min immersion of graphene in ITO solution) graphene on copper after air drying. **c** ITO nanoparticle-deposited graphene on SiO_2_ (300 ± 10 nm)/Si substrate before annealing. **d** ITO nanoparticle-deposited graphene on SiO_2_ (300 ± 5 nm)/Si substrate after vacuum annealing at 450 °C for 1 h. **e** Optical microscopic imagery of ITO nanoparticle-decorated graphene on SiO_2_ (300 ± 5 nm)/Si substrate after annealing. The black dots represent ITO nanoparticles

The morphologies of the as-prepared graphene on Cu and air-dried graphene on Cu after 1-min immersion in ITO solution are shown in Fig. 2a, b, respectively. As depicted in Fig. 2a, no black dots and island/flake nature were detected, demonstrating that the continuous high-quality graphene was grown free of amorphous carbon.

As shown in Fig. 2b, the ITO nanoparticles were obviously dispersed on the surface of graphene, and were adsorbed into the graphene during the immersion period of graphene in ITO solution. ITO nanoparticle-decorated graphene on SiO_2_/Si before and after annealing at 450 °C for 1 h under vacuum is shown in Fig. 2c, d. Highly aggregated nanoparticle clusters were observed instead of individual nanoparticles in the before-annealing sample, as shown in Fig. 2c. As depicted in Fig. 2d, the ITO forms a uniform distribution of individual sphere-shaped nanoparticles with diameters in the range 25–35 nm. Some nanoparticles attached to each other, with diameters of 100–150 nm, were randomly distributed and consisted of tiny ITO nanoparticles on graphene. The interaction of nanoparticles with graphene is the main factor for understanding the growth mechanism of a nanostructure on graphene. In fact, the approach used is simple and versatile, whereby the thermodynamic and kinetic mechanisms are involved in the adsorption and nucleation process of nanoparticles on the graphene surface [38]. In general, the adsorption energies (*E*_a_, representing the strength of the interaction between the adsorbed particle and graphene) and diffusion barriers (Δ*E*) are the key kinetic barriers controlling the growth morphology of nanoparticles on graphene, and the adsorbed nanoparticle transport process is governed by the surface diffusivity, obeying the Arrhenius law [39, 40]. The particle size and density are mainly governed by Δ*E* for the adsorbed particle on graphene because Δ*E* represents the rate at which the adsorbed particle can contact the nearest preexisting particle island before being attracted by other adsorbed particles to form a new particle island. As In is the main element in ITO, with a high *E*_a_ (15.15 kcal mol^−1^) and low Δ*E* (1.68 kcal mol^−1^), it plays the main role in the adsorption and growth of nanoparticles on graphene, because much lower Δ*E* for In on graphene tends to produce a much lower density of nanoparticles with a much higher particle size [40]. It is therefore very important to maintain a short immersion time of graphene in the ITO solution. However, if the immersion time is sufficiently long for aggregation of nanoparticles, the nanoparticles will crack, and their quality will be degraded during the annealing period. Furthermore, adsorbed nanoparticles introduce dipole interaction, indirect electron and magnetic interaction, and elastic interaction with the system, which play important roles in the nucleation and surface morphology during growth. In and Sn have large electric dipole moments and, owing to the dipole–dipole interaction, the two types of nanoparticles undergo strong repulsion, which increases the barrier energy for condensation of the adsorbed particles [39]. Although graphene is hydrophobic in nature, it can also show hydrophilic behavior, depending on many factors, such as defects and functionalization owing to the high surface free energy of these factors [41–43]. Therefore, the morphology of nanoparticles on graphene also depends on the regions of high surface free energy within the graphene. The existence of ITO nanoparticle-decorated CVD graphene on SiO_2_/Si can be observed in the optical microscopy image (a damaged graphene area was specifically selected) as shown in Fig. 2e.

**Fig. 3:**
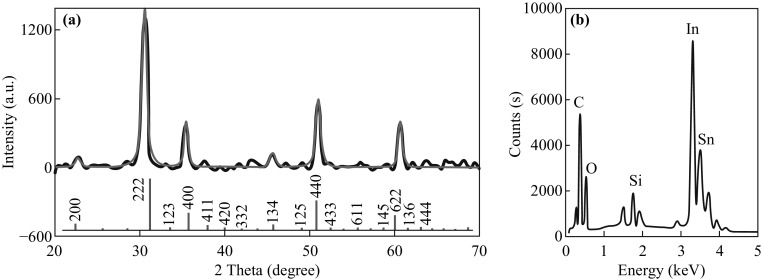
**a** XRD pattern of ITO nanoparticles deposited on graphene after annealing at 450 °C for 1 h under vacuum (black curve) and Lorentzian fitting curve (red curve). **b** EDX spectroscopy of ITO nanoparticles deposited on graphene after annealing at 450 °C for 1 h under vacuum. (Color figure online)

An XRD pattern of the ITO nanoparticle-decorated graphene, as shown in Fig. 3a, after annealing at 450 °C for 1 h under vacuum reveals that the nanostructure was synthesized by this method on graphene, corresponding to typical ITO with a crystallite cubic structure according to obvious reflections from the characteristic lattice planes of (211), (222), (400), (431), (440), and (622) observed in the Lorentzian fitting curve (red curve) at 22.67°, 30.52°, 35.41°, 45.62°, 51.00°, and 60.64°, respectively (JCPDS card No. 06-0416). No peaks corresponding to the crystalline phases of Sn were detected, indicating that a solid solution of Sn in In_2_O_3_ was present in the synthesized ITO nanoparticles on graphene [30, 33, 44]. Moreover, an interplanar spacing 2.924 Å corresponding to (222) was obtained, which agrees well with the interplanar spacing of (222) in the JCPDS card. EDX analysis spectroscopy, as shown in Fig. 3b, was conducted to identify the composition of the ITO nanoparticle-decorated graphene and revealed the presence of approximately 10 wt% of Sn in the ITO nanoparticles on graphene. The doping level of Sn strongly affects the electrical properties of ITO, whereas the Sn atoms cannot be effectively substituted for the In atom sites after a desired level of Sn doping is reached. Additionally, as reported previously, the ITO containing 10 wt% Sn exhibited excellent electrical performance [45, 46]. The peaks relevant to C and Si obtained from EDX originated from graphene and the SiO_2_/Si substrate. Table 1 shows the bulk chemical composition of In and Sn of the samples.

**Table 1:** Bulk chemical composition of In and Sn

Element	wt%	at%
Indium (In)	87.19	72.28
Tin (Sn)	9.42	7.56

**Fig. 4:**
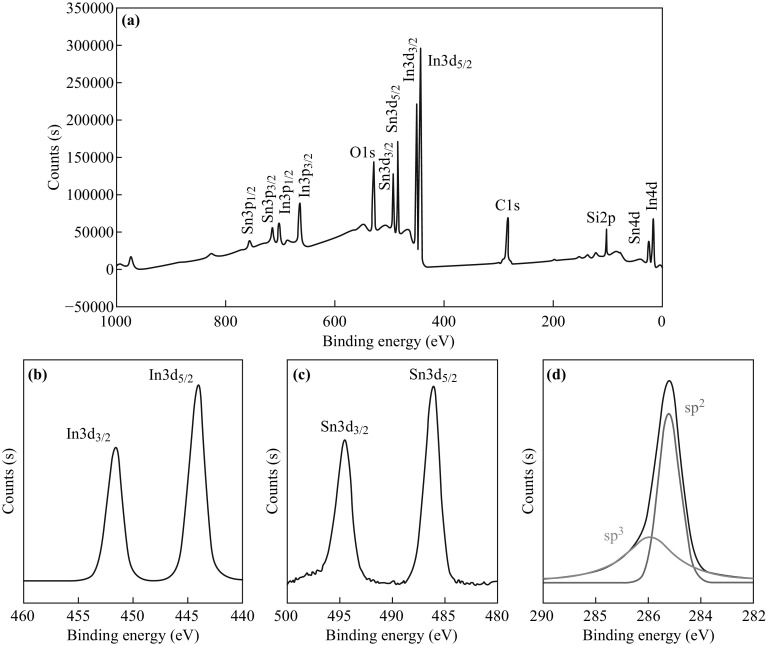
XPS of ITO nanoparticles deposited on graphene after annealing at 450 °C for 1 h under vacuum: **a** wide-scan spectra, **b** narrow-scan spectra of In3d, **c** narrow-scan spectra of Sn3d, and **d** narrow-scan spectra of C1 s

As depicted in Fig. 4a, the XPS wide-scan spectra of ITO nanoparticle-decorated graphene after annealing are shown to further explore the elemental composition and the quality of the samples. In3d_3/2_ and In3d_5/2_ peaks were observed at 452.1 and 444.31 eV, respectively, as shown in the narrow-scan XPS spectra of In3d in Fig. 4b. In3d_5/2_ shows a 0.71 ± 0.2 eV chemical shift from the position corresponding to In3d_5/2_ of metallic In (443.6 ± 0.2 eV). Sn3d_3/2_ and Sn3d_5/2_ peaks were observed at 494.82 and 486.83 eV, respectively, as shown in the narrow-scan XPS spectra of Sn3d in Fig. 4c. Sn3d_5/2_ shows a 2.33 ± 0.2 eV chemical shift from the position corresponding to Sn3d_5/2_ of metallic Sn (484.5 ± 0.2 eV). The chemical shift to a higher binding energy of both peaks indicated that the ITO nanoparticles deposited on graphene were free of metallic compounds, and that only the oxide forms of In and Sn were present [46–48]. The lack of a peak at approximately 485.6 eV validated that no reduction of Sn^iv^ to Sn^ii^ occurred during the synthesis of the ITO solution and annealing, because the electrical conductivity of ITO decreased with the reduction of Sn^iv^ to Sn^ii^ [47]. An O1 s peak was observed at approximately 530.93 eV; two peaks may be fitted to O1 s, because the O^2−^ lattice ions in ITO have two possible locations in the lattice, namely the oxygen-deficient and nondeficient regions [49]. Figure 4d shows the XPS C1 s narrow spectrum deconvoluted into two peaks. The lower-binding-energy peak located at approximately 285.11 eV corresponds to the C–C *sp* ^2^ bonding of graphene, whereas the high-binding-energy peak located at approximately 285.94 eV corresponds to *sp* ^3^ C–C bonding. This *sp* ^3^ C–C bonding carbon was mainly due to contamination [47, 50]. The existence of vacancies and interstitials such as defects in graphene also promoted *sp* ^3^ C–C bonding.

### Effect of ITO Nanoparticles and Annealing on Raman Signatures of Graphene

**Fig. 5:**
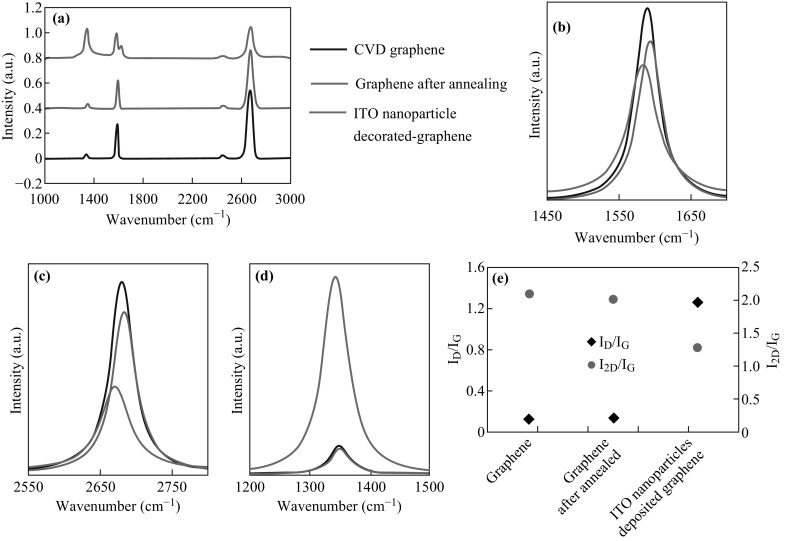
**a** Raman spectrum of CVD graphene (excitation wavelength 532 nm) after transference to SiO_2_ (300 ± 5 nm)/Si (black curve), after vacuum annealing at 450 °C for 1 h (red curve) and ITO nanoparticles-decorated graphene (blue curve), zoomed spectra of Lorentzian-fitted graphs of the **b** G peak, **c** 2D peak, and **d** peak. **e** *I*_D_/*I*_G_ and *I*_2D_/*I*_G_ ratios of graphene under different situations. (Color figure online)

The crystallization of amorphous ITO nanoparticles starts at 160–180 °C. However, above 400 °C, annealing is required to complete crystallization with high electrical performance [51, 52]. It is critical to understand the key factor to enhance the quality of graphene with respect to the defects even after annealing (450 °C in vacuum for 1 h) during the fabrication of samples. Figure 5a shows the Raman spectra of CVD graphene (black curve), CVD graphene after annealing (red curve), and ITO nanoparticle-decorated CVD graphene (blue curve). As shown by the red curve in Fig. 5a, no increment in the D peak intensity was observed, even after vacuum annealing at 450 °C for 1 h, indicating that graphene was stable at the employed temperature and vacuum conditions. This is because graphene did not react with active gases in vacuum annealing, creating no defects in graphene, such as carbon–oxygen *sp* ^3^ bonds and vacancies [53]. G and 2D peaks were observed in the annealed graphene at 1593.19 and 2684.12 cm^−1^, respectively, with a small D peak at 1349.68 cm^−1^. However, the G and 2D peaks of graphene after vacuum annealing at 450 °C for 1 h revealed small blueshifts of 3.985 and 4.155 cm^−1^, respectively, as shown by the red curves of Fig. 5b, c with respect to the G and 2D peaks of CVD graphene (black curves of Fig. 5b, c).

The blueshift of the G and 2D bands was due to the hole-doping effect, because other adsorbates on CVD graphene were much more reduced during annealing, introducing adsorption sites on graphene for atmospheric molecules such as H_2_O and O_2_ after exposure to atmospheric air [53, 54]. The 2D peak revealed two different responses depending on the type of doping, whereby the hole doping introduces the blueshift and the electron doping introduces the redshift, mainly due to the charge-transfer-induced modification of the graphene equilibrium lattice parameter [54, 55]. Three separate disorder-induced peaks, a high-intensity D peak that scatters from the *K* to *K*′ intervalley, a D′ peak that scatters from the *K* to *K* intervalley, and a low-intensity D + G combination scattering peak were observed at 1342.00, 1621.08, and 2933.42 cm^−1^, respectively, as shown in the blue curve in Fig. 5a after the ITO nanoparticles were deposited on graphene. Meanwhile, the D′ and D + G peaks developed with a dramatically increased D peak, validating that the degree of disorder in graphene increases with the introduction of ITO nanoparticles on graphene, owing to the interaction of ITO nanoparticles with the graphene lattice. The reduction in the intensities of the G and 2D peaks was observed with respect to undoped graphene (black curve), which indicates the surface coverage of graphene by ITO nanoparticles. Interestingly, on average, the D, G, and 2D band wavenumbers revealed redshifts of 5.65, 5.69, and 9.74 cm^−1^, respectively, after ITO nanoparticle deposition, as shown by the blue curve in Fig. 5b–d. Electron/hole doping or *n*-type/*p*-type doping in graphene introduced a red/blueshift in the Raman spectrum, and the magnitude of the shift and relative 2D band intensity with respect to undoped graphene depended on the dopant concentration [56]. Graphene subjected to tensile/compressive strain also strongly affected the red/blueshift of the Raman spectrum owing to the elongation of the C–C bond [2, 10, 57]. The redshift Raman spectrum in the ITO nanoparticle-decorated graphene revealed that the ITO nanoparticles introduced an n-type doping effect with lattice distortion on the graphene. Furthermore, the strain between the ITO nanoparticles and graphene more strongly affected the Raman shift than the carrier density modification of graphene, because the redshift of the 2D band was higher than that of the G band (the shift of the 2D band was approximately 1.7 times stronger than that of the G band) [58].

Figure 5e shows the *I*_D_/*I*_G_ and *I*_2D_/*I*_G_ variation with different conditions of graphene. Graphene after annealing at 450 °C for 1 h revealed *I*_D_/*I*_G_ and *I*_2D_/*I*_G_ values of 0.13 and 2.01, respectively, which are practically identical to those for the CVD graphene (0.122 and 2.09, respectively); this demonstrated that vacuum annealing at 450 °C for 1 h had no significant effect on the structural changes and creation of new defects in graphene. However, after the deposition of the ITO nanoparticles on graphene, an increment in *I*_D_/*I*_G_ of up to 1.254 and the reduction in *I*_2D_/*I*_G_ to 1.277 were revealed, indicating that disorder-induced structural changes of graphene occur as the result of the introduction of ITO nanoparticles over graphene.

### Electrical and Optical Properties

**Fig. 6:**
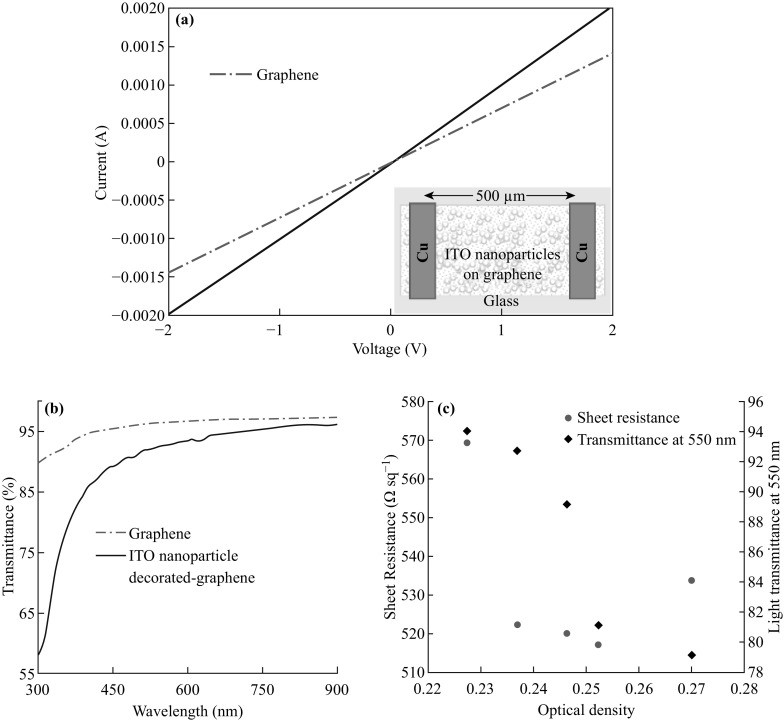
**a** Voltage–current (*V*–*I*) characteristics of graphene and ITO nanoparticles-deposited graphene (inset image shows the schematic diagram of the prepared sample). **b** UV–Vis spectrum of graphene and ITO nanoparticle-deposited graphene. **c** Variation in sheet resistance and light transmittance as functions of optical density

Figure 6a shows the voltage–current (*V*–*I*) characteristics of graphene (dotted line), the ITO nanoparticle-deposited graphene (continuous line) at room temperature, and a schematic diagram of the prepared samples. The plot illustrates the dependence of the current, through the 500 µm length of the sample, on voltage, applied across the sample by using Cu electrodes. The reciprocal gradients of the graphs represent the corresponding resistance (*R*) between the two conductors, whereas the high slope of the graph indicates a lower resistance. The high slope of the *V*–*I* curve according to the ITO nanoparticles-deposited graphene implied a lower resistance than that of graphene. The mean sheet resistance of the ITO nanoparticle-decorated graphene was approximately 522.21 Ω sq^−1^ (standard deviation, SD = 2.53 Ω sq^−1^), obtained using the four-point probe method, where the decrease in sheet resistance was approximately 28.2% relative to CVD graphene, with a mean sheet resistance of 727.43 Ω sq^−1^ (SD = 15.54 Ω sq^−1^). The ITO nanoparticles introduce electron doping to graphene, which improves the surface electron density of graphene, because the conductivity is proportional to the carrier concentration, resulting in a lower sheet resistance from the ITO nanoparticle-deposited graphene. However, the conductivity of the ITO nanoparticle-deposited graphene was not as high as that of the continuous ITO film, owing to the discontinuous nature of the ITO nanoparticles over graphene. Figure 6b shows the UV–Vis transmittance spectra of CVD graphene (dotted line) and ITO nanoparticle-decorated graphene (continuous line). As expected, the light transmittance of graphene was reduced after the introduction of ITO nanoparticles over graphene, owing to the light absorption of the ITO nanoparticles. However, the ITO nanoparticle-decorated graphene is highly transparent in the visible region (more than 85%).

The density of ITO nanoparticles can affect electrical and optical properties of the hybrid materials. The nanoparticle density is directly proportional to optical density, and according to the Beer–Lambert law of absorbance, the density of nanoparticles can be represented by the optical density owing to the constant absorptivity coefficient and path length for each ITO/graphene hybrid sample [59, 60]. Figure 6c reveals the variation in sheet resistance and optical transmittance at 550 nm wavelength of the hybrid with optical density. Owing to the linear relationship between particle density and optical density, the light transmittance decreases with increasing optical density. Simply put, the higher the optical density, the higher the transmittance, because optical density represents how well a material can block a particular wavelength of light. The light transmittance of the hybrid reveals an inverse relationship with the density of ITO nanoparticles in the hybrids [59]. The sheet resistance increases with decreasing density of ITO nanoparticles. This behavior of light transmittance and sheet resistance with nanoparticle density can be explained as follows. A higher density of nanoparticles implies a high surface coverage of graphene, which absorbs a high amount of light when light passes through the material. At the same time, a high density of ITO nanoparticles can increase the electron density of graphene, thereby improving the electrical conductivity of the hybrid. However, up to a certain optical density value, the sheet resistance of the hybrid reduced, after which the value increased, as revealed by the red dots of the graph. The nanoparticles become aggregated beyond a certain value of nanoparticle density, which can easily crack and degrade the quality of the nanoparticles during annealing, to the detriment of the electrical properties of the hybrid [51]. For optoelectronics applications, it is very important to maintain high light transmittance and low sheet resistance; in this study, the best results of light transmittance and sheet resistance were obtained for an optical density value of the hybrid of 0.2369.

## Conclusions

In this study, a novel, simple, and versatile method was proposed to deposit ITO nanoparticles on graphene. This method involved a combination of an environmentally friendly solution-based electroless deposition approach and subsequent vacuum annealing. Graphene was grown by the CVD method, and an ITO solution was synthesized by the organic-additive-free aqueous sol–gel method with economical salts of In(NO_3_)_3_^•^H_2_O and SnCl_4_. It was observed that a unique structure with 25–35 nm evenly dispersed crystallized ITO nanoparticles was formed on the graphene. The functional properties of the ITO nanoparticles–graphene hybrid transparent conducting electrode were demonstrated to be good and reproducible with a mean sheet resistance of 522.21 Ω sq^−1^, representing an improvement of 28.2% relative to that of the CVD graphene, and showed more than 85% light transmittance in the visible region. The annealing conditions did not significantly affect the Raman signals. However, the ITO nanoparticles induced the redshifting of the entire Raman signatures, in which D, G, and 2D peaks were redshifted by 5.65, 5.69, and 9.74 cm^−1^, respectively. The unique architecture of the ITO nanoparticles on graphene with a high surface area, enhanced electrical conductivity, and good optical properties in the visible range renders such hybrids suitable for optoelectronic and electrocatalytic applications.
